# Dietary and supplemental long-chain omega-3 fatty acids as moderators of cognitive impairment and Alzheimer’s disease

**DOI:** 10.1007/s00394-021-02655-4

**Published:** 2021-08-15

**Authors:** Amy H. R. Wood, Helen F. Chappell, Michael A. Zulyniak

**Affiliations:** Nutritional Epidemiology Group, School of Food Science and Nutrition, University of Leeds, Leeds, LS2 9JT UK

**Keywords:** Polyunsaturated fatty acids, Docosahexaenoic acid, Eicosapentaenoic acid, Alzheimer’s disease, Neurodegeneration, Cognitive impairment

## Abstract

**Purpose:**

There is an ever-growing body of literature examining the relationship between dietary omega-3 polyunsaturated fatty acids (ω3 PUFAs) and cerebral structure and function throughout life. In light of this, the use of ω3 PUFAs, namely, long-chain (LC) ω3 PUFAs (i.e., eicosapentaenoic acid and docosahexaenoic acid), as a therapeutic strategy to mitigate cognitive impairment, and progression to Alzheimer’s disease is an attractive prospect. This review aims to summarise evidence reported by observational studies and clinical trials that investigated the role of LC ω3 PUFAs against cognition impairment and future risk of Alzheimer’s disease.

**Methods:**

Studies were identified in PubMed and Scopus using the search terms “omega-3 fatty acids”, “Alzheimer’s disease” and “cognition”, along with common variants. Inclusion criteria included observational or randomised controlled trials (RCTs) with all participants aged ≥ 50 years that reported on the association between LC ω3 PUFAs and cognitive function or biological markers indicative of cognitive function linked to Alzheimer’s disease.

**Results:**

Evidence from 33 studies suggests that dietary and supplemental LC ω3 PUFAs have a protective effect against cognitive impairment. Synaptic plasticity, neuronal membrane fluidity, neuroinflammation, and changes in expression of genes linked to cognitive decline have been identified as potential targets of LC ω3 PUFAs. The protective effects LC ω3 PUFAs on cognitive function and reduced risk of Alzheimer’s disease were supported by both observational studies and RCTs, with RCTs suggesting a more pronounced effect in individuals with early and mild cognitive impairment.

**Conclusion:**

The findings of this review suggest that individuals consuming higher amounts of LC ω3 PUFAs are less likely to develop cognitive impairment and that, as a preventative strategy against Alzheimer’s disease, it is most effective when dietary LC ω3 PUFAs are consumed prior to or in the early stages of cognitive decline.

**Supplementary Information:**

The online version contains supplementary material available at 10.1007/s00394-021-02655-4.

## Dementia, Alzheimer’s disease, and cognitive impairment

Encompassing a collection of syndromes, dementia is characterised by progressive impairment of memory, language, behaviour and visuospatial function, leading to compromised independence [1]. It is estimated to affect 50 million people globally [2] and is caused by a number of diseases that trigger irreversible damage to cells in the brain. The most common of these diseases is Alzheimer’s disease (AD), representing 60% to 80% of dementia cases [3]. No treatment has yet been found to prevent or cure cognitive impairment or its progression to dementia and AD [3].

Cognitive impairment, especially difficulty retaining new information, is an early marker of AD. Numerous clinical tests have been validated to evaluate different aspects of cognition that can identify individuals at increased risk of AD within a few years (e.g., rate of learning over time, immediate recall, and delayed recall) [4]. In addition to memory and recall, episodic assessments can be used to assess specific neurocognitive functions: problem‐solving, planning, language, attention, and visuospatial skills. The diagnosis of cognitive impairment and elevated risk of AD is defined by guidelines set by the National Institute on Aging and the Alzheimer’s Association [4]. The guidelines offer flexible assessment criteria to match the assessor’s expertise (general practitioner vs specialist), accessibility to diagnostic resources (e.g., image scanning and blood biomarkers), and the cognitive challenges presented by the patient. In light of this flexibility, there is acknowledgment that the AD population is not homogenous, but a heterogeneous population of subgroups defined by their cognitive abilities and diagnostic criteria [5]. The effect of age on cognitive impairment is undeniable; however, it is important to evaluate the effect of modifiable environmental factors, such as diet, both on the prevention of cognitive decline and onset of AD. Indeed, identifying cardinal risk factors that commonly contribute to early cognitive impairment and risk of AD, could help (i) identify individuals at high risk of cognitive impairment, (ii) project an individual’s progression toward AD, and (iii) inform strategies to delay the onset of AD.

## PUFAs as mediators of cognitive impairment

There is general agreement across studies and study design, of an association between healthy diets, cognitive function, and risk of AD [6–9]. That is, prudent and ‘Mediterranean’ diets, characterised by greater intake of whole grains, fresh fruits and vegetables, fish, and polyunsaturated fatty acids, associate with reduced risk of cognitive impairment, compared to ‘Western’ diets characterised by processed foods, saturated fats, refined grains, and added sugars [10]. PUFAs, lipidic molecules defined by the presence of more than one double bond in the aliphatic chain, are typically more abundant in healthy ‘whole food’ diets, such as those consumed in prudent and ‘Mediterranean’ diets. The two main series of PUFAs are omega-3 (ω3): α-linolenic acid (ALA, C18:3), docosahexaenoic acid (DHA, C22:6) and eicosapentaenoic acid (EPA, C20:5); and omega-6 (ω6): linoleic acid (LA, C18:2) and arachidonic acid (ARA, C20:4) [11].

Precursor ω6 and ω3 PUFAs, LA and ALA, are essential fatty acids determined by diet as they cannot be endogenously synthesised by humans [12], while long-chain (LC) PUFAs (e.g., EPA, DHA, and ARA) may be either endogenously synthesised from their precursor ω3 or ω6 PUFA or consumed directly through diet or supplementation.

ALA, found in high concentrations in chia seeds, walnuts, and oils of flaxseed, canola and soybean [13], can be synthesised by enzymatic desaturation and elongation to supply the body with EPA and DHA [14]. Delta-6 desaturase, the enzyme required in the first rate-limiting stage of ALA conversion [11], is also implicated in the metabolism of LA, meaning that both PUFAs compete for desaturation [13]. The rate of ALA conversion is, therefore, generally low at less than 5% [15]. Food sources of EPA and DHA notably consist of fish and seafood products, including fish oils [16]. Mean intakes of EPA and DHA through food in the UK have been estimated at 244 mg/day [17], substantially lower than the recommended 450 mg/day as stipulated by the Scientific Advisory Committee on Nutrition [18]. However, PUFA intake is perhaps best predicted by the ω6:ω3 ratio present in the diet, rather than the intake of a single PUFA. While ratios between 1:1 and 5:1 have demonstrated positive effects in ameliorating general disease burden [19], the modern Western diet, characterised by a high consumption of meat, vegetable oils, and processed foods, is often reported to have an ω6:ω3 in excess of 15:1 [20]. The chronic low-grade inflammation induced by this aspect of diet may have consequences for neuronal health, generating an environment that impairs cognitive function and fosters AD pathology [21]. Structurally, ω3 and ω6 LC PUFAs comprise around 30–35% of fatty acids in the brain [11] and have been shown in pre-clinical and human studies to exert effects on metabolic processes involved in brain development and function throughout the life course [11, 22, 23] through modulation of membrane fluidity, gene expression, and inflammation.

### Membrane fluidity

The fatty acid composition of cell membranes affects the membrane fluidity index by influencing membrane packing [11]. The abundance of LC PUFAs within the phospholipid fractions of the brain, predominantly DHA and ARA [14], increases the fluidity of neuronal membranes [12], which in turn modulates the function of transmembrane and peripheral proteins, such as receptors, enzymes and ion channels involved in vital cellular processes [11]. Following a decrease in absorption across the blood–brain barrier and changes to fatty acid metabolism, the aging brain sees a decrease in membrane LC PUFA concentration [12] and a corresponding decline in membrane fluidity—an observation documented in patients with clinically diagnosed cognitive impairment and AD [24, 25]. Interestingly, aged-related membrane rigidity has been reversed in the hippocampus of pre-clinical AD models through a DHA-enriched diet [26] but its effect on cognitive function in individuals already diagnosed with AD is inconsistent [27, 28].

### Gene expression

LC PUFAs are implicated in the expression of several genes [29]. Targets for modulation are primarily from the nuclear receptor superfamily, namely, the retinoid X receptor, retinoic acid receptor and peroxisome proliferator-activated receptor (PPAR) [22]. They function as ligand-activated transcription factors in retinoid signalling [29], which are associated with synaptic plasticity, subsequent memory and learning ability [22] and, therefore, AD disease pathologies [30]. Interestingly, LC ω3 PUFAs show a greater potency in modifying nuclear receptor gene expression compared to LC ω6 PUFAs [31]. PPAR can bind to multiple fatty acids and their derivatives; however, PUFAs, in particular EPA, show superior binding affinity compared to ω6 PUFAs and saturated fatty acids [32]. Furthermore, increased ω3 intake may increase transthyretin [29], a thyroid transport protein that acts as a scavenger of β-amyloid (Aβ) protein fragments [14]. The aggregation and deposition of Aβ^42^ fragments as neuropathological plaques is considered a primary hallmark of AD [33]. A human model of AD has shown that over-expression of transthyretin supresses Aβ plaque formation, putatively through sequestration [34], while pre-clinical AD models suggest a causal pathway connecting decreased dietary ω6:ω3 ratios with increased PPAR signalling and improved cognitive measures [35, 36]. Collectively, this positions modification of dietary LC PUFAs as a viable approach to improve cognitive function in AD; however, heterogeneity of effect sizes in human trials [37] challenges the generalisability of results.

### Inflammation

Eicosanoids are a group of inflammatory mediators that include prostaglandins, thromboxanes and leukotrienes [38, 39]. PUFAs act as precursors to the synthesis of eicosanoids through metabolism by cyclooxygenase (COX) and 5-lipoxygenase (5-LOX) enzymes [22]. ARA, a 20-carbon LC ω6 PUFA, is in plentiful supply in neural cell membranes, making it the primary substrate for the majority of eicosanoid production [38]. ARA-derived eicosanoids, which include the 2-series of prostaglandins and thromboxanes, as well as the 4-series of leukotrienes [22], potentiate autocrine and paracrine inflammation [40], with in vitro evidence suggesting they induce Aβ plaque formation [31]. Conversely, EPA, a 20-carbon LC ω3 PUFA, competes with ARA for COX and 5-LOX enzymes [41], thereby lessening ARA metabolism and levels of ARA-derived eicosanoids [38, 42]. In addition, EPA is a precursor for lesser pro-inflammatory eicosanoids, namely, the 3-series of prostaglandins and thromboxanes, and the 5-series of leukotrienes [22, 43], and (along with DHA) anti-inflammatory resolvins and neuroprotectins [38]. Of particular interest is neuroprotectin D1, which can inhibit COX2 expression, reduce ARA metabolism [44], and modulate Bcl-2 expression towards an anti-apoptotic state; thereby, ameliorating the deleterious effects of inflammation [45].

In summary, evidence from molecular, epidemiological, and preclinical and human studies elucidates dietary LC ω3 PUFAs as mediators of cognitive decline and risk of AD; however, a summary of current literature is required to critique and evaluate the strength of existing evidence linking dietary LC ω3 PUFAs with cognitive decline and explain the heterogeneity observed in study results. By understanding the primary mechanisms that contribute to the development and progression of cognitive decline and AD, we are well positioned to offer an evaluation of this evidence. In doing so, we aim to determine the role of dietary and supplemented LC ω3 PUFAs as moderators of cognitive decline and risk of AD.

## Method

PubMed and Scopus databases were searched, using “omega-3 fatty acids”, “Alzheimer’s disease” and “cognition” as key search terms, as well as variants of these (“n-3 fatty acids, “PUFAs”, “AD”). Inclusion criteria were: (i) original randomised controlled trials (RCTs) or observational studies; of (ii) human participants ≥ 50 years; (iii) investigating the association between dietary or supplemented ω3 PUFAs; on (iv) qualitative or biological assessment of cognitive function; (v) as a marker of AD risk. Exclusion criteria were: (i) in vitro or (ii) preclinical studies, and (iii) non-original or (iv) non-peer-reviewed publications. No exclusion regarding initial publication date was set and all studies published up to March 2020 were eligible. Identified studies were then compiled into tables to assess and compare the participant sample profile, methods, and outcomes in a clear and concise manner (see Tables 1, 2).

**Table 1:** Observational studies investigating the association between dietary ω3 intake and AD risk

References	Cohort	Year	Study characteristics	Follow-up	Dietary recall	Findings
[46]	Rotterdam Study	1997	*n* 5386 Age: ≥ 55 years	1.2 years	Semi-quantitative FFQ	Fish consumption (> 18.5 g/d) associated with a 70% reduced risk of AD without cerebrovascular disease (95% CI: 0.1–0.9)
[47]		2009	*n* 5395 Age: ≥ 55 years	9.6 years		Total fish and ω3 PUFA intake not associated with long-term AD risk
[48]	Cardiovascular Health Cognition Study	2005	*n* 2233 Age: ≥ 65 years	5.4 years	FFQ	Consumption of fatty fish showed 41% decreased risk of AD in those without ApoE ɛ4 allele (95% CI: 0.36–0.95)
[49]	Chicago Memory and Aging Project	2003	*n* 815 Age: 65–94 years	3.9 years	FFQ	Consumption of one or more fish meal per week reduced risk of AD by 60% (95% CI: 0.2–0.9). Total ω3 PUFA and DHA intake significantly and linearly associated with a reduced risk of AD
[50]	Hordland Health Study	2007	*n* 2031 Age: 70–74 years	Cross-sectional	FFQ	Consumption of lean and fatty fish associated with better scores on five of six cognitive tests compared to no consumption. Fish oils only associated with improved scores in one of six tests
[51]	Framingham Heart Study	2006	*n* 899 Age: 55–88 years	9.1 years	Semi-quantitative FFQ	Significant 47% reduction in risk of developing all-cause dementia in upper quartile of plasma DHA content (mean DHA intake = 0.18 g/d, mean fish intake = 3 servings/week) (95% CI: 0.29–0.97). No significant reduction in risk of AD specifically
[55]	Rush Memory and Aging Project	2015	*n* 923 Age: 58–98 years	4.5 years	Semi-quantitative FFQ	Moderate and high adherence to MIND diet showed lower risk of AD compared to first tertile. High adherence to DASH and Mediterranean diet also associated with lower AD risk
[57]		2016	*n* 915 Mean age: 81.4 years	4.9 years		Intake of food sources of ω3 PUFAs not associated with cognitive decline, however, fish oil supplement consumers had slower rates of decline in global cognition and episodic memory measures than non-consumers

*FFQ* food frequency questionnaire

**Table 2:** Clinical trials investigating the association between supplementary ω3 intake and cognitive function

References	Study design	Year	Sample	Diagnostic approach	Dose and method of supplementation	Exposure period	Outcome Measures	Findings
[58]	Randomised double-blind placebo-controlled trial (OmegAD)	2006	Mild to moderate AD patients *n* 174 Mean age: 74	Clinical diagnosis: DSM-IV, medical history, psychometric testing, blood analyses, MRI	1700 mg/day DHA and 600 mg/day EPA as capsules Placebo: corn oil capsules	12 months	MMSE, ADAS-cog	No statistically significant difference in cognition between groups. A subset with very mild cognitive decline showed a significant decrease in rate of cognitive decline
[59]		2015	AD patients *n* 174 Mean age: 74					Significant positive association between changes in plasma DHA and decrease in cognitive decline rate. Plasma EPA associated with slower decline rate based on several ADAS-cog parameters. No associations with level of AD
[80]		2009	Mild to moderate AD patients *n* 35 Mean age: 70			6 months	Inflammatory markers in plasma (IL-6, TNF-α, sIL-1RII) and cerebrospinal fluid (tau, hyperphosphorylated tau, Aβ_42_)	No significant effect on biomarkers
[81]		2014	Moderate AD patients *n* 40 Mean age: 70.5				Change in levels of F2-isoprostane, 8-iso-PGF2α and 15-keto-dihydro-PGF2α	No significant effect on biomarkers
[84]		2013	Mild to moderate AD patients *n* 174 Mean age: 75			12 months	Transthyretin in plasma and cerebrospinal fluid as indicated by nephelometry	Significant increase in plasma transthyretin, non-significant increase in cerebrospinal fluid transthyretin
[60]	Randomised double-blind placebo-controlled trial	2008	Mild to moderate AD or MCI *n* 29 Mean age: 75.1	DSM-IV interview and medical assessment by psychiatrist or neurologist	720 mg/day DHA and 1080 mg/day EPA as capsules Placebo: olive oil capsules	5.5 months	CIBIC, ADAS-cog	Improvement in general clinical function but not cognitive function. Significant improvement in ADAS-cog score in ω3 MCI group compared to placebo, but not observed in AD group
[28]	Randomised double-blind placebo-controlled trial	2017	MCI *n* 219 Mean age: 74.5	Clinical diagnosis by neurologist: Petersen’s criteria MCI, medical history, NINCDS-ADRDA criteria for AD incidence	2000 mg/day DHA derived from algae as capsules Placebo: soybean oil capsule	12 months	Chinese version of the WAIS-R, MRI	Increased hippocampal volume and significant improvement in scores for Full Scale Intelligence Quotient, Information and Digit Span for intervention group compared to placebo
[61]		2018	MCI *n* 217 Mean age: 73.6			24 months		Significant improvement in scores for Full Scale Intelligence Quotient, Verbal Intelligence Quotient, Information and Digit Span for intervention group compared to placebo
[27]	Randomised double-blind placebo-controlled trial	2010	Mild to moderate AD *n* 295 Mean age: 76	Alzheimer’s Disease Cooperative Study clinic institutional review boards	2000 mg/day DHA derived from algae as capsules Placebo: corn or soy oil capsules	18 months	ADAS-cog, sum-of-boxes CDR, rate of brain atrophy	No effect on any measures of cognitive decline
[62]	Randomised double-blind placebo-controlled trial	2010	Age-related cognitive decline *n* 485 Mean age: 70		900 mg/day DHA as capsules Placebo: corn and soy oil	5.5 months	CANTAB paired associate learning, Verbal Recognition Memory, Pattern Recognition Memory, Stockings of Cambridge, Spatial Working Memory, MMSE	Two-fold increase in plasma DHA levels that correlated with significantly fewer PAL errors and was associated with improved immediate and delayed Verbal Recognition Memory
[63]	Randomised double-blind placebo-controlled trial	2012	MCI *n* 35 Mean age: 64.9	Neuropsychological assessment by clinical psychologists	1300 mg/day DHA and 450 mg/day EPA derived from fish as capsules Placebo: corn oil capsules	12 months	Neuropsychological battery comprised of components from: WMS-R, RAVLT, WAIS-R, CDT and WAIS-III	Significant improvement in short-term and working memory, immediate verbal memory, delayed recall capability and change in memory over 12 months
[64]	Randomised placebo-controlled trial	2006	MCI, organic brain lesions, AD *n* 39 Mean age: 64.9	Petersen’s criteria assessed by authors	240 mg/day ARA and 240 mg/day DHA as capsules Placebo: olive oil capsules	3 months	Japanese version of the RBANS test	Significantly improved immediate memory in MCI and organic lesions compared to placebo, not in AD
[65]	Randomised double-blind placebo-controlled trial	2015	CIND, early AD *n* 76 Mean age: 71.1	Medical and neuropsychological history from memory clinic referral, NINCDS-ADRDA criteria, MRI, blood analyses	625 mg/day DHA and 600 mg/day EPA as capsules Placebo: olive oil capsules	4 months	MMSE Serial Sevens, MMSE World Backwards, immediate, delayed and recognition verbal memory	No significant effects observed in any cognitive function measures
[66]	Randomised placebo-controlled superiority trial (MAPT)	2017	Memory complaints *n* 1680 Mean age: 75.3	Medical history from general practitioner, MMSE	800 mg/day DHA and 225 mg/day EPA as capsules Placebo: paraffin oil capsules	36 months	Battery of tests including free and total recall of Free and Cued Selective Reminding Test, 10 items on MMSE, COWAT, Digit Symbol Substitution Test, Category Naming Test, CDR, TMT	No significant effect on composite score with or without combination with multi-domain intervention
[67]		2018						No significant effect on battery test score
[71]		2017	Lowest quartile of ω3 index Memory complaints *n* 183 Mean age: 76					ω3 supplementation group showed reduced decline in COWAT scores compared to placebo. No significant difference in scores for other tests, although all scores lower in intervention group
[68]	Double-blind placebo-controlled trial (Alpha Omega Trial)	2012	No cognitive impairment *n* 2911 Mean age: 69.1	N/A	160 mg/day DHA and 240 mg/day EPA as margarine treated with fish oil, with or without 2000 mg ALA Placebo: standard margarine	40 months	MMSE	No significant effect on MMSE score
[69]	Randomised double-blind placebo-controlled trial	2015	No cognitive impairment, adult macular degeneration *n* 3424 Mean age: 72.7	N/A	350 mg/day DHA and 650 mg/day EPA as capsules Placebo: standard AREDS formulation	60 months	Composite scores for: TICS-M, letter fluency, category fluency, alternating fluency, WMS-III, Backward Counting, delayed recall of TICS-M and WMS-III	No significant effect on composite scores
[70]	Double-blind placebo-controlled trial	2016	No cognitive impairment *n* 44 Age: 50–70	N/A	880 mg/day DHA and 1320 mg/day EPA derived from fish as capsules Placebo: sunflower oil capsules	6 months	Object Location Memory	Significant increase in OLM scores observed in supplementation group compared to placebo
[72]	Randomised double-blind placebo-controlled trial	2014	Mild to moderate cognitive impairment *n* 199 Mean age: 74.6	12-month follow-up and screening prior to intervention using DSM-IV, MMSE	180 mg/day DHA and 120 mg/day EPA as cod liver oil capsule Placebo: coconut oil capsule	6 months	MMSE, AMT	No significant effect on MMSE or AMT scores
[73]	Randomised double-blind placebo-controlled trial	2010	Mild AD *n* 225 Mean age: 73.7	NINCDS-ADRDA criteria, MMSE, MRI	1200 mg/day DHA and 300 mg/day EPA as Fortasyn Connect nutrition combination in Souvenaid drink Placebo: isocaloric drink	3 months	WMS-R delayed verbal recall task, ADAS-cog, CIBIC	Fewer reduced scores and significantly more improved scores in WMS-R compared to placebo
[74]	Randomised double-blind placebo-controlled trial	2017	Prodromal AD *n* 311 Mean age: 71	NINCDS-ADRDA criteria, CSF, MRI, ^18^F fluorodeoxyglucose PET analysis by clinician	1200 mg/day DHA and 300 mg/day EPA as Fortasyn Connect nutrition combination in Souvenaid drink Placebo: isocaloric drink	24 months	Modified version of Neuropsychological Test Battery	No significant effect on composite score, although cognitive decline much lower than expected
[78]	Randomised placebo-controlled trial	2014	Probable AD *n* 39 Mean age: 75.9	NINCDS-ADRDA criteria, MMSE, CDR	675 mg/day DHA and 975 mg/day EPA derived from fish as capsules; or 675 mg/day DPA, 975 mg/day EPA derived from fish plus 600 mg/day alpha lipoic acid as tablet ω3 placebo: soybean oil as capsule with 5% fish oil	12 months	Change in levels of F2-isoprostane, MMSE, ADL/IADL, ADAS-cog	No significant difference in isoprostane levels. Effects on cognition more significant with addition of LA than omega-3 s alone
[82]	Randomised double-blind placebo-controlled trial	2017	MCI *n* 13 Mean age: 66.5	Mayo clinic criteria for MCI by neurology specialist	880 mg/day DHA and 1320 mg/day EPA as capsules Placebo: sunflower oil capsules	6 months	Cerebral perfusion as indicated by cerebral blood flow and cerebral blood volume	Increase in cerebral blood flow and volume larger in omega-3 intervention than placebo group
[83]	Open study^a^	2015	MCI, AD *n* 29 Mean age: 77.4	NINCDS-ADRDA criteria, Petersen’s criteria for MCI, MMSE	1000 mg/day DHA and 1000 mg/day EPA as Smartfish drink (also contained 10ug vitamin D3 and resveratrol)	17 months	Aβ phagocytosis (as indicated by flow cytometry and microscopy), transcription of inflammatory genes (as indicated by RC-PCR), resolvin-D1 production (as indicated by enzyme immunoassay), MMSE	Aβ phagocytosis increased significantly in MCI but not in AD. No other significant associations

*DSM-IV* diagnostic and statistical manual of mental disorders fourth edition, *NINCDS-ADRDA* National Institute of neurological and communicative disorders and stroke and the Alzheimer’s disease and related disorders association

^a^Not classified as a clinical trial but was included in the table for the purpose of comparison to other biomarker studies

## Results

Eight observational studies and 25 RCTs were selected for review (see Supplementary Fig. 1).

### Observational studies

The data from observational studies generally support an inverse association between dietary LC ω3 PUFA intake and cognitive decline and risk of AD (Table 1). The Rotterdam Study (1997) is the earliest account in this review of the link being demonstrated in a population-based cohort of elderly subjects (≥ 55 years). Data from this study showed a relative risk (RR) of AD at 0.3 (95% CI: 0.1–0.9) with daily fish consumption above 18.5 g [46]. Interestingly, a follow-up study in 2009 reported contrasting results [47]. Fish, total ω3, EPA and DHA intake of 5395 subjects did not associate with AD [47]. The 10-year follow-up period may have meant that baseline dietary information was not representative of the participants’ diets throughout the duration of the study; however, this does not rule out the potential relevance of earlier dietary habits in AD development.

Further studies have reported similar results [48–51], including a smaller cohort used in the Chicago Memory and Aging Project (*n* 815), wherein consumption of one or more fish meals per week generated a RR of 0.4 (95% CI: 0.2–0.9), adjusted for age, sex, race, education, energy intake and presence of the ApoE ɛ4 polymorphism [49]. Although this result was near borderline significance (*p* = 0.07), the inverse, linear association between total ω3 PUFA intake and AD showed clear significance (*p* = 0.01) [49]. This relationship was also seen for DHA intake specifically, with those in the top three quintiles of intake showing multivariate-adjusted RR as low as 0.2 (95% CI: 0.1–0.8) [49]. As less than 1% of the cohort reported taking ω3 PUFA supplements, it can be deduced that this intake was primarily achieved through diet.

A later prospective study by Schaefer et al.[51] analysed baseline plasma DHA in participants of the Framingham Heart Study and found that those in the upper quartile (mean 3 servings of fish/week) had an all-cause dementia RR of 0.53 (95% CI: 0.29–0.97) compared to those in the lower three quartiles. Although of similar magnitude, the association between plasma DHA and AD was not significant (RR = 0.61; 95% CI: 0.31–1.18; *p*= 0.14) when upper and lower quartiles were compared [51]. The dietary intake of DHA was also high for these subjects in the cohort, at 1800 mg/day. Fish intake was significantly associated with plasma DHA; however, once adjusted for plasma DHA, the association between dietary fish and DHA intake and AD was not significant [51]. This suggests that DHA is the primary component of fish effecting AD risk and underlines the importance of DHA bioavailability and absorption into the blood, which can be improved by co-ingestion of a high fat meal alongside supplement use [52]. In addition, variability of fish DHA content (% fatty acids) between species and global region [53, 54], is likely to contribute to the heterogeneity of results we observed between observational studies and suggests that subgrouping fish intake by its DHA content may help to reduce this heterogeneity.

A more recent community-based cohort study, the Rush Memory and Aging Project, identified a decreased risk of AD in participants with the highest adherence scores to MIND (Mediterranean-DASH Intervention for Neurodegenerative Delay), DASH (Dietary Approaches to Stop Hypertension) and Mediterranean diets, with hazard ratios (HR) at 0.47 (95% CI: 0.26–0.76), 0.61 (95% CI: 0.38–0.97) and 0.46 (95% CI: 0.26–0.79), respectively [55]. In particular, the MIND diet showed a HR of 0.65 (95% CI: 0.44–0.98) even with moderate adherence [55]. In addition to adequate intake of fish, the diet consists of 15 ‘brain-healthy’ food aspects, including increased fruit and vegetables and reduced red meat and butter, which make it difficult to deduce the role of LC ω3 PUFAs in ameliorating AD risk from these results. These findings agree with previous work investigating the combined effects of multi-nutritional LC ω3 PUFAs-rich drinks [56] on cognitive function and AD risk and encourage future studies to identify novel LC ω3 PUFA-nutrient interactions or nutritional patterns that might offer even greater protection against cognitive impairment and future AD risk.

The same cohort was tested for cognitive ability using a 21-test battery to assess global cognition and five specified cognitive domains, including episodic memory and visuospatial ability [57]. Although ω3 PUFAs from food sources were not associated with cognition, ω3 supplementation was significantly associated with slower rates of decline in global cognition and episodic memory scores compared to non-consumers [57]. This suggests that an increased habitual intake of ω3 PUFAs reliably delivers an adequate dosage to influence cognitive outcomes. Several of these studies also identified a significant relationship between increased ALA consumption and reduced AD risk, but interestingly only in ApoE ɛ4 carriers [49, 57].

In summary, observational studies support the notion that LC ω3 PUFAs offer a protective effect against cognitive decline and risk of AD. The period of study in observational studies is far longer than can be realistically achieved in a clinical setting, meaning that prolonged exposure to dietary LC ω3 PUFAs can be represented in analysis, in comparison to a transient response to increased LC ω3 PUFA intake. However, the observational studies identified in this review were conducted on European and American populations, meaning that these data may not be generalisable to other populations with varying ratios of ApoE polymorphism, and differing quantities and sources of fish and LC ω3 PUFA consumption.

Future epidemiological studies are required to evaluate the distinct and interactive associations between LC ω3 PUFAs and foods they are commonly consumed alongside (i.e., as in a Mediterranean diet) and the effect of cooking methods on the efficacy of LC ω3 PUFAs. Concurrent dietary parameters such as increased fruit and vegetable intake and decreased saturated fat intake may also play a role in cerebral health and support the metabolic effects of LC ω3 PUFAs.

### Clinical trials

#### Cognitive testing

The OmegAD trial [58] was the first large, randomised placebo-controlled trial to investigate the effects of ω3 PUFAs on the cognitive ability of AD patients (Table 2). Subjects were randomised to receive either ω3 PUFA capsules (1700 mg/day DHA and 600 mg/day) for the full 12 months of the study, or 6 months of placebo capsules followed by 6 months of ω3 PUFA capsules. Scores for the Mini-Mental State Examination (MMSE) and the cognitive portion of the Alzheimer’s Disease Assessment Scale (ADAS-cog) were taken at baseline, 6 and 12 months; however, no statistically significant differences in scores between the two groups were established [58]. A subgroup of 32 patients with very mild AD (MMSE > 27 points) showed a significant attenuation of cognitive decline compared to placebo, as indicated by MMSE score after 6 months of intervention [58].

A further study as part of the OmegAD trial found that the diminution of cognitive decline as measured by ADAS-cog scores was significantly associated with increasing plasma DHA, and that plasma EPA was associated with a number of parameters within the ADAS-cog [59]. Similar conclusions can be drawn from a shorter trial by Chiu et al. [60], assessing a formulation with lower DHA and higher EPA content than the OmegAD trial (DHA:EPA 2:3 [60] vs 3:1 [58]). Although the mixed model indicated a positive change in global clinical function in the intervention group, as measured by Clinician's Interview-Based Impression of Change (CIBIC), the improvement of ADAS-cog score over the intervention was selective to those with mild cognitive impairment (MCI); no effect was seen in AD patients. Intriguingly, this study reported lower levels of plasma ARA in the ω3-treated group compared to the control [60], which may suggest a decrease in pro-inflammatory eicosanoid synthesis caused by the inhibitory action of EPA on ARA metabolism. Increased EPA on erythrocyte membranes was associated with improved ADAS-cog scores [60].

Zhang et al. [28] investigated the effect of high dose algal-derived DHA on cognitive function of those with MCI, defined as subjective memory complaints accompanying a score of 1.5 standard deviations below age- and education-matched controls in MMSE memory subtest, but without the presence of AD or related diseases [28]. Following 12 months of supplementation with 2000 mg DHA/day, the intervention group achieved significantly higher test scores for Full Scale Intelligence Quotient and subdomains of Information and Digit Span [28]. In a follow-up paper after 24 months of supplementation, the authors reported the same significant improvements as seen after 12 months, with an additional improvement in Verbal Intelligence Quotient [61]. This dosage has also been trialled in patients with mild to moderate AD (MMSE 14–26), but there was no significant effect on rate of cognitive decline as indicated by ADAS-cog and Clinical Dementia Rating (CDR) [27]. It may be important to note that one of the placebos used in these studies was soybean oil, known to contain, amongst other fatty acids, a significant amount of ALA [13], which may have compromised the estimate of the effect size. Indeed, as a precursor for LC ω3 PUFA, it is likely that a small percentage of ALA was synthesised into EPA or DHA and contaminated the control group; however, without another control group, the magnitude of contamination cannot be determined.

The efficacy of LC ω3 PUFA supplementation on MCI has been further investigated by a number of trials [62–65]. Yurko-Mauro et al. [62] examined DHA supplementation in subjects with age-related cognitive decline (MMSE > 26). After 24 weeks of treatment (900 mg/day), the intervention group had significantly fewer Paired Associate Learning pattern errors than placebo subjects, as well as improved immediate and delayed Verbal Recognition Memory [62]. These changes were significantly associated with increased plasma DHA levels, of which the intervention group exhibited a twofold rise in plasma DHA [62]. Improved immediate verbal memory has also been reported in MCI patients treated with DHA-EPA combination supplements [63] and DHA supplements at a substantially lower dosage [64]. Interestingly, this study [64] delivered 240 mg/day DHA alongside an equivalent dose of ARA. Increased concentration of ARA in neuronal membranes may have increased the fluidity index of brain cells in the treated group, consequently leading to improved synaptic function. By contrast, AD subjects did not exhibit any improvement in immediate memory [64]. Similar findings were documented in another trial [65]; however, this study also found no association between LC ω3 PUFA supplementation (625 mg/day DHA 600 mg/day EPA) and cognitive function in cognitively impaired individuals without dementia (CIND), contradicting evidence from other research [62–64]. These studies raise questions regarding the effects of study parameters, such as study duration and dosage, that may have contributed to the results of trials successfully associating LC ω3 PUFAs with improved cognition in MCI. Specifically, they suggest that durations of ≥ 5 months and DHA supplements exceeding 900 mg/day, are required to elicit a significant effect on cognitive ability in elderly populations.

Several large-scale trials (*n* ~ 1600–3400), including the Multidomain Alzheimer Preventive Trial (MAPT) and Alpha Omega Trial, have found no association between LC ω3 PUFAs and cognition, both in those with memory complaints [66, 67] and those deemed cognitively healthy [68, 69]. Although these studies use large sample sizes and substantial intervention periods, the dosage of LC ω3 PUFAs are comparatively lower than in other trials conducted in this area (Table 2). While these levels better mimic the level of ω3 PUFAs achievable through the diet, the results of studies using higher supplementary doses have more consistently reported significant effects of LC ω3 PUFAs on cognitive function in MCI [28, 61, 63] and at-risk older individuals [70]. Interestingly, a secondary analysis of MAPT study subjects with memory complaints in the lowest quartile of ω3 index revealed an increase in Controlled Oral Word Association Test (COWAT) scores for the intervention group over the 36-month intervention, where the control group experienced a decrease in mean score [71]. Although this was the only finding to achieve statistical significance, the intervention group performed better than the placebo group in all other tests conducted [71]. Interestingly, a smaller 6-month trial (*n* 199) supports these findings in cases of MCI, reporting no significant change in MMSE or Abbreviated Mental Test (AMT) following ω3 PUFA supplementation [72].

Scheltens et al. [73] randomised patients with mild AD (MMSE 20–26) to receive either 1200 mg/day DHA and 300 mg/day EPA as a medical supplement drink (Souvenaid), or an isocaloric placebo drink, for 12 weeks. A significant improvement was reported in revised Wechsler Memory Scale (WMS-R) delayed recall score, although ADAS-cog and CIBIC scores did not change. A subgroup analysis of patients with very mild AD also achieved significantly improved immediate verbal recall after 12 weeks [73]. A 24-month trial on a similar population with prodromal AD (mean age 71, mean MMSE 26.7) has since been conducted to establish the longer term influence of Souvenaid [74]. The authors reported a moderately positive effect based on lower rates of cognitive decline in the intervention group than were expected based on projected 24-month decline observed in AD patients [75]. However, no significant effect on the composite score of the neuropsychological test battery used was reported [74]. Souvenaid contains a combination of other nutrients including vitamin C (80 mg) and a number of B-vitamins (3 μg B12, 1 mg B6, 400 μg folic acid). Interactions between B-vitamins and ω3 PUFAs in affecting reduced rates of brain atrophy have been reported in MCI [76]. Although the efficacy of B-vitamin treatment in AD is still yet to be thoroughly established [77], the potential for the effects observed in the Souvenaid trials to be influenced by the additional nutrients in the drink make it difficult to isolate the impact of ω3 PUFAs alone.

The Souvenaid trials raised questions about the combination of ω3 PUFAs with other agents in supplementation. Shinto et al.[78] analysed the effects of ω3 PUFA treatment (675 mg/day DHA 975 mg/day EPA) with and without the addition of α-lipoic acid (600 mg/day), an organosulphur compound and antioxidant implicated in the protection of mitochondria from oxidative damage [79] and reduction of inflammatory markers [78], as is seen in AD. Although no significant difference was found in ADAS-cog scores, Instrumental Activities of Daily Living (IADL) scores of probable AD subjects showed significantly less decline over the 12-month trial period for both ω3 and ω3-α-lipoic acid groups compared to placebo; however, a significant difference between MMSE scores for treatment and placebo groups was only observed in the case of combined ω3-α-lipoic acid supplementation [78]. As levels of endogenous α-lipoic acid decrease with age [79], these results suggest the use of lipoic acid in conjunction with ω3 PUFA supplementation may have biological plausibility for cases of late-onset AD, although further research is warranted. In the placebo arm, the authors used a capsule containing soybean oil and 5% fish oil [78]. While the ω3 concentrations may not have been as high as in the intervention arm, the potential influence on results is worth noting.

#### Biomarkers

As well as cognitive testing, numerous studies also measured biological changes as indicators of cognitive function [78, 80–84]. As part of the OmegAD study, 35 AD subjects were randomised to 6-month supplementation of ω3 PUFAs (1700 mg/day DHA 600 mg/day EPA) or placebo [80]. Plasma samples were tested at baseline and 6 months for the inflammatory markers IL-6, tumour necrosis factor-alpha (TNF-α) and soluble interleukin-1 receptor type-II (sIL-1RII). Cerebrospinal fluid was extracted by lumbar puncture at baseline and 6 months and tested for IL-6, TNF-α, sIL-1RII, as well as tau protein, hyperphosphorylated tau protein and Aβ peptides, all hallmarks of AD pathology [33]. A significant association between sIL-1RII and Aβ_42_ was made at baseline; however, no associations related to ω3 PUFAs were found with any of the biomarkers measured [80]. The same sample was examined for changes in urinary F2-isoprostanes and prostaglandin F2_α_, both formed through COX pathways associated with oxidative stress [81]. 15-keto-dihydro-PGF2_α_, a metabolite of prostaglandin F2_α_, was used as a biomarker of inflammation. No changes were observed in any of these biomarkers. Further research on ω3 PUFA supplementation and F2-isoprostane levels in cases of probable AD reached a similar conclusion [78]. However, neither study reported a qualitative assessment of cognitive function at baseline and follow-up, so it is not possible to confirm the null hypothesis or validate the association between biomarkers and cognitive function.

A number of biomarker studies have reported associations between ω3 PUFAs and quantitative indicators of cognitive function [82, 83]. A cerebral perfusion study revealed increased cerebral blood flow and volume in posterior corticoid regions of MCI subjects following high dose ω3 PUFA supplementation (880 mg/day DHA and 1320 mg/day EPA) for 6 months [82]. Cerebral hypoperfusion has been associated with AD as a consequence of neuronal tissue damage and brain atrophy [85]. Although no statistically significant effect of 18-month high dose DHA supplementation was reported in slowing the rate of brain atrophy in cases of mild to moderate AD [62], a more recent study [61] of similar design with a larger sample size (*n* 219 vs 102 [62]) identified an association between the same DHA dosage (2000 mg/day) and increased hippocampal volume in MCI subjects after 12 months [61]. In another study, administration of a drink containing 1000 mg DHA and 1000 mg EPA amongst other nutrients, including vitamin D and resveratrol (Smartfish), was significantly associated with increased Aβ phagocytosis by monocytes as indicated by flow cytometry in MCI and pre-MCI subjects, but not in AD [83]. These studies further support the efficacy of LC ω3 PUFAs as early interventions and suggest that physical brain measures, while time-consuming and costly, provide a more reliable measure of cognitive degeneration compared to blood and cerebrospinal fluid analytes.

The largest biomarker study identified in this review, with more than 100 subjects, was undertaken by Faxén-Irving and colleagues, as a further part of the OmegAD trial [84]. Transthyretin, a Aβ binding protein thought to reduce plaque formation [86], was the compound of interest. 174 patients with mild to moderate AD completed two consecutive 6-month supplementation periods as two groups—ω3/ω3 and placebo/ω3. Plasma transthyretin levels after 6 months significantly decreased in the placebo group, at which point a correlation was identified between DHA and transthyretin (rho = 0.17, *p* = 0.03) [84]. At the 12-month follow-up, after both groups had been supplemented with ω3 PUFAs, plasma transthyretin increased significantly in both groups, at which point a correlation was identified between MMSE scores and transthyretin (rho = 0.16, *p* = 0.03), alongside an inverse correlation with ADAS-cog scores (rho = − 0.2, *p* = 0.02) [84]. Cerebrospinal fluid was analysed over 6 months in a subset of 35 subjects; however, the increase in cerebrospinal fluid transthyretin observed in the ω3/ω3 treatment group was not significant [84]. This trial is one of few that offers evidence that ω3 PUFAs may improve cognition in those with established AD.

In controlled trials, participants have usually already received a diagnosis of AD, meaning that ω3 PUFAs are tested for their treatment effect on a disease that has already been established with notably manifested symptoms of memory impairment. Indeed, based on the data reviewed here, wherein the majority of clinical trials that made positive associations between supplementation and cognition were conducted in cases of MCI as opposed to AD, a protective effect of ω3 PUFAs is inferred.

## Conclusion

Cognitive impairment and AD present major challenges for clinical medicine, with multi-faceted pathologies that are still not entirely understood. The role of environmental factors to prevent cognitive impairment and subsequent AD continues to be an area of extensive research, particularly pertinent to late-onset cases. Due to their widely recognised influence on cognitive function, ω3 PUFAs present a compelling opportunity for nutritional therapy in cognitive decline and AD.

Evidence from observational studies appears promising for prevention of cognitive decline. Regular consumption of LC ω3-PUFA rich foods in healthy populations without pre-existing AD or dementia suggests a protective effect against future AD. Conversely, clinical trials that primarily focus on participants already diagnosed with AD consistently report no effect. The incongruence between these findings highlights the precedence of LC ω3 PUFA intake earlier in life, before cognitive decline is apparent and AD risk has been recognised.

Although many observational studies adjusted for known confounders, including the ApoE ɛ4 allele, this was not consistent across the literature. Those that specifically analysed ApoE ɛ4 suggest that the metabolic effects of different LC ω3 PUFAs differ between ɛ4 carriers and non-carriers; therefore, more targeted studies within this high-risk population are warranted to better understand ɛ4-specific effects of different types of LC ω3 PUFAs.

Overall, LC ω3 PUFA supplementation appears to be well-tolerated, even in high doses (Table 2). Although LC ω3 PUFAs may not be a validated treatment strategy for cognitive impairment or AD based on the current evidence, their successful implementation as an early intervention strategy for neuroprotection in MCI and healthy older populations is encouraging. Large-scale trials of long duration (≥ 5 months) using higher dosage supplements (900 mg DHA/day) across diverse populations are required to validate these findings, to improve generalisability and determine safe and optimum dosage. Furthermore, the combination of LC ω3 PUFAs with other nutrients of interest, such as α-lipoic acid and B-vitamins, is an intriguing area of research that may identify additional supplementation strategies and nutrient interactions to prevent and ameliorate cognitive decline in the elderly.

## Supplementary Information

Below is the link to the electronic supplementary material.

Supplementary file1 (DOC 58 KB)

## Abbreviations

5-LOX: 5-Lipoxygenase
Aβ: Amyloid-beta peptide
AD: Alzheimer’s disease
ADAS-cog: Alzheimer’s disease assessment scale cognitive subscale
ADL: Activities of daily living
ALA: Alpha-linolenic acid
AMT: Abbreviated mental test
ApoE: Apolipoprotein E
ARA: Arachidonic acid
CDR: Clinical dementia rating
CDT: Clock-drawing test
CIBIC: Clinician’s interview-based impression of change
COWAT: Controlled oral word association test
COX: Cyclooxygenase
DHA: Docosahexaenoic acid
EPA: Eicosapentaenoic acid
HVLT-DR: Hopkins verbal learning test with delayed recall
IADL: Instrumental activities of daily living
IL-6: Interleukin-6
LA: Linoleic acid
MCI: Mild cognitive impairment
MMSE: Mini-mental state examination
PPAR: Peroxisome proliferator-activated receptor
LC PUFAs: Long-chain polyunsaturated fatty acids
RAVLT: Rey auditory verbal learning test
RBANS: Repeatable battery for assessment of neuropsychological status
sIL-1RII: Soluble interleukin-1 receptor type-II
TICS-M: Modified telephone interview for cognitive status
TMT: Trail making test
TNF-α: Tumour necrosis factor-alpha
WAIS-III: Wechsler adult intelligence scale third edition
WAIS-R: Revised wechsler Adult intelligence scale
WMS-R: Revised wechsler memory scale
ω3 PUFAs: Omega-3 polyunsaturated fatty acids
ω6 PUFAs: Omega-6 polyunsaturated fatty acids
