# Supplementary material for: Dietary and supplemental long-chain omega-3 fatty acids as moderators of cognitive impairment and Alzheimer’s disease
Source: Eur J Nutr. 2021 Aug 15;61(2):589–604. doi: 10.1007/s00394-021-02655-4 (PMC8854294; doi:10.1007/s00394-021-02655-4)
Supplement: Supplementary file 1 — Supplementary file1 (DOC 58 KB) [file 394_2021_2655_MOESM1_ESM.doc]

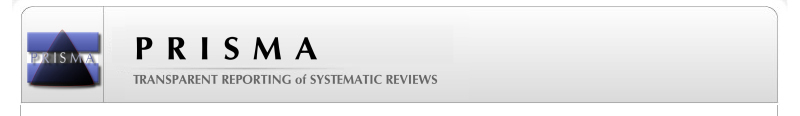
**PRISMA 2009 Flow Diagram**

**Screening**

**Included**

**Eligibility**

**Identification**

Records identified through database searching
(n = 713)

Additional records identified through other sources
(n = 0)

Records screened
(n = 713)

Duplicates and Records excluded

(n=672)

(n = )

Full-text articles assessed for eligibility
(n = 41)

Full-text articles excluded, with reasons
(n = 8)

Studies included in qualitative synthesis

(Review)
(n = 33)
